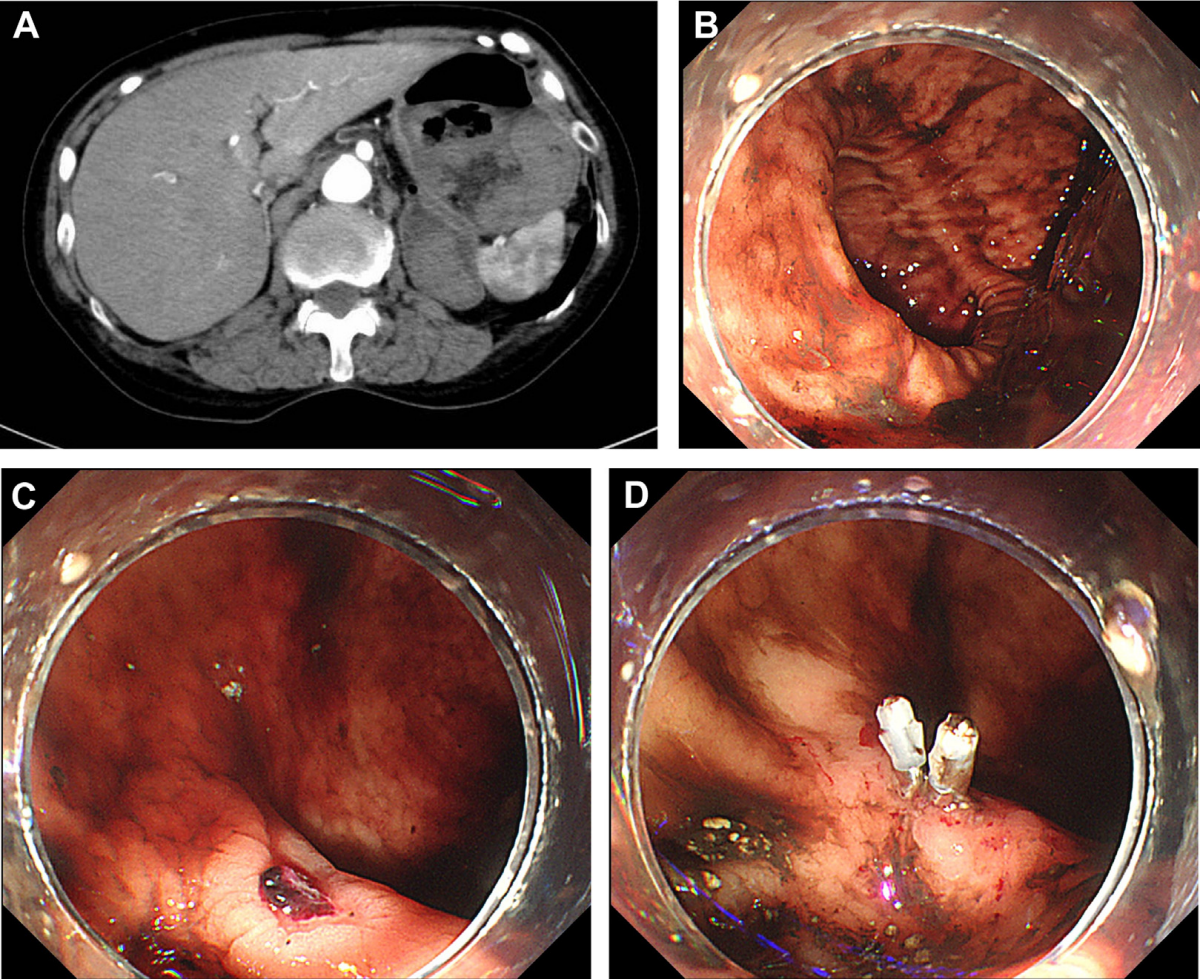# Successful Clip Hemostasis for Ulcer Bleeding in a Large Gastric Diverticulum

**DOI:** 10.1016/j.gastha.2022.10.010

**Published:** 2022-10-27

**Authors:** Kento Shionoya, Akiko Sasaki, Chihiro Sumida

**Affiliations:** Shonan Gastroenterology Medicine Center, Shonan Kamakura General Hospital, Kamakura, Kanagawa, Japan

A 71-year-old female visited our hospital owing to fainting and black stools. Subsequent laboratory testing revealed a low hemoglobin level of 9.6 g/dL. A sac structure sized 60 mm on the posterior cardia and fluid retention in the stomach was observed with contrast-enhanced abdominal computed tomography as a high-density area (Figure A). Emergency upper gastrointestinal endoscopy revealed a gastric diverticulum with an oval entrance 6.7 cm in diameter on the posterior cardia and a small ulcer bleeding at the bottom of the diverticulum (Figure B and Figure C). Clip hemostasis was performed on a visible bleeding vessel on the ulcer surface (Figure D). Second-look endoscopy confirmed successful hemostasis. The patient did not experience further bleeding and was discharged. She had been prescribed methotrexate for rheumatoid arthritis, which may have caused the gastric diverticulum.

Gastric diverticulum is a rare disorder that generally affects the posterior wall of the fundus owing to the anatomically thin muscle layer and the penetration of large blood vessels. However, surgical hemostasis is sometimes required because of the difficulty in detecting the bleeding site and the risk of perforation due to endoscopic procedures. In our case, endoscopic hemostasis was successfully achieved with reliable visibility of the bleeding site.

**Figure:**